# Can Brain Waves Really Tell If a Product Will Be Purchased? Inferring Consumer Preferences From Single-Item Brain Potentials

**DOI:** 10.3389/fnint.2019.00019

**Published:** 2019-06-28

**Authors:** Nobuhiko Goto, Xue Li Lim, Dexter Shee, Aya Hatano, Kok Wei Khong, Luciano Grüdtner Buratto, Motoki Watabe, Alexandre Schaefer

**Affiliations:** ^1^Department of Psychology, Kyoto Notre Dame University, Kyoto, Japan; ^2^School of Business, Monash University Malaysia, Bandar Sunway, Malaysia; ^3^Cognitive Neuroscience, Institute of Neuroscience and Medicine (INM-3), Jülich Research Center, Jülich, Germany; ^4^Department of Psychology, Monash University Malaysia, Bandar Sunway, Malaysia; ^5^Kochi University of Technology, Kami, Japan; ^6^Japan Society for the Promotion of Science, Tokyo, Japan; ^7^School of Marketing, Faculty of Business and Law, Taylor’s University Malaysia, Subang Jaya, Malaysia; ^8^Institute of Psychology, University of Brasília, Brasília, Brazil

**Keywords:** attention, preferences, decision-making, EEG, event-related potentials, consumer behavior, motivational relevance, neuromarketing

## Abstract

Recent research has shown that event-related brain potentials (ERPs) recorded while participants view lists of different consumer goods can be modulated by their preferences toward these products. However, it remains largely unknown whether ERP activity specific to a single consumer item can be informative about whether or not this item will be preferred in a shopping context. In this study, we examined whether single-item ERPs could reliably predict consumer preferences toward specific consumer goods. We recorded scalp EEG from 40 participants while they were viewing pictures of consumer goods and we subsequently asked them to indicate their preferences for each of these items. Replicating previous results, we found that ERP activity averaged over the six most preferred products was significantly differentiated from ERP activity averaged across the six least preferred products for three ERP components: The N200, the late positive potential (LPP) and positive slow waves (PSW). We also found that using single-item ERPs to infer behavioral preferences about specific consumer goods led to an overall predictive accuracy of 71%, although this figure varied according to which ERPs were targeted. Later positivities such as the LPP and PSW yielded relatively higher predictive accuracy rates than the frontal N200. Our results suggest that ERPs related to single consumer items can be relatively accurate predictors of behavioral preferences depending on which type of ERP effects are chosen by the researcher, and ultimately on the level of prediction errors that users choose to tolerate.

## Introduction

There has been in recent years a growing interest in the integration of cognitive neuroscience methods with consumer behavior (CB) research. This trend has led to the development of a discipline often termed “Neuromarketing” or “Consumer Neuroscience” (Ariely and Berns, 2010; Javor et al., 2013; Plassmann et al., 2015; Hakim and Levy, 2018). This new field of knowledge has often been linked to a number of controversial claims, such as the contention that brain activity measured while consumers view a product could be used to infer attitudes and intentions toward it, or the purported existence of a “buy button” in the brain (Ariely and Berns, 2010). Nevertheless, scientific research on the neural correlates of CB has yielded valuable results, especially regarding the question of the neural substrates of preferences for consumer goods, a trend of research that has mainly used functional magnetic resonance imaging (fMRI) (e.g., Knutson et al., 2007; Levy et al., 2011; Falk et al., 2012; see also Bartra et al., 2013).

A handful of studies have also approached the question of CB prediction using human electrophysiological (EEG) techniques and, in particular, the event-related potentials (ERP) method (Junghöfer et al., 2010; Jones et al., 2012; Khushaba et al., 2012; Boksem and Smidts, 2015; Pozharliev et al., 2015; Telpaz et al., 2015; Bosshard et al., 2016; Schaefer et al., 2016; Goto et al., 2017; Ma et al., 2018), an approach that has several advantages: It is non-invasive and harmless; it is significantly less costly than fMRI; and the vast existing body of ERP literature enables relatively robust links between ERP components and their underlying cognitive processes (Goto et al., 2017). Interestingly, EEG/ERP methods have been widely used by private Neuromarketing consultancy companies (Morin, 2011; Boksem and Smidts, 2015; Stanton et al., 2017; Hakim and Levy, 2018).

Existing published ERP research on CB has led to the discovery of strong relationships between indices of preferences for consumer goods and mainly three ERP components: the N200, the late positive potential (LPP) and positive slow waves (PSW) (Pozharliev et al., 2015; Telpaz et al., 2015; Schaefer et al., 2016; Goto et al., 2017; Ma et al., 2018). The N200 is an early brain potential occurring approximately between 180 and 400 ms post-stimulus onset with a largely fronto-central topography. The N200 has been associated with an automatic allocation of attentional resources to goal- or task-relevant stimuli (Carretié et al., 2004; Olofsson et al., 2008; Kanske and Kotz, 2010; Sass et al., 2010; Walker et al., 2011) or with the detection of prediction errors and conflict monitoring (Yeung et al., 2004; Baker and Holroyd, 2011) and it is often associated with medial prefrontal areas (Yeung et al., 2004). The LPP is a positive deflection often observed between 400 and 800 ms with centro-parietal maxima, which is believed to reflect an overt form of attentional engagement toward relevant stimuli. It is often observed in reaction to visual information conveying emotional contents (Schupp et al., 2006; Codispoti et al., 2007; Walker et al., 2011; Liu et al., 2012). In a combined EEG-fMRI study, Liu et al. (2012) found that the LPP obtained in response to emotional pictures was linked to heightened activity in visual cortices, the prefrontal cortex and the amygdala. The PSW is a late positive deflection often observed after 800 ms, which can extend up to 3 seconds after the onset of a visual stimulus (Foti and Hajcak, 2008; Goto et al., 2017). It is often observed at both fronto-central and parietal sites and it is thought to reflect a more sustained form of processing that may involve working memory (WM) processes (García-Larrea and Cézanne-Bert, 1998; Rämä et al., 2000; Watts et al., 2014). Overall, the observation that these ERPs are linked to consumer preferences has been interpreted as a reflection of *Motivated Attention* processes; which can be defined as selective attention toward motivationally relevant information. More specifically, the association of these ERPs with consumer preferences might reflect different stages of attention toward preferred items. These processes may include an initial automatic allocation of attentional resources followed by an overt form of attentional engagement, and more sustained attentional processes involving WM (Junghöfer et al., 2010; Goto et al., 2017; see also Lang et al., 1997, and Vuilleumier and Huang, 2009 for a wider theoretical perspective).

However, two important outstanding questions remain, which the current study aimed to address. First, most published studies have reported results that rely on the analysis of neural activity related to groups of different goods rather than single-item neural activity (i.e., brain activity related to a single given object). In other words, the dominant approach has been to obtain ERP waveforms by averaging EEG activity related to viewing several products (e.g., a DVD, a chocolate box from brand X, a book, a tablet), instead of isolating ERP waveforms specific to one product (e.g., a chocolate box from brand X). This approach creates an obstacle in the translation of neuroimaging methods into market research applications, for which clear product-specific inferences are often necessary. In other words, the claim that neural activity related to single items can predict preferences toward them remains largely untested from the perspective of classical ERP research methods. For the remainder of this article, we will refer to neural activity related to single items as “single-item” (SI) activity, and activity obtained through averaging brain activity over different items as “group-related” activity.

The second outstanding question relates to a more classical approach of “group-related” ERPs related to consumer preferences. Within this approach, the debate is still open regarding which specific brain potentials are related to consumer preferences. For instance, Telpaz et al. (2015) found effects of preference for consumer goods only on the N200, and reported that similar effects on the P300 potential were not significant. In addition, they did not report any further effects on late positivities (LPP or PSW). Using independent component analysis and a mobile EEG approach, Roberts et al. (2018) found evidence suggesting that a P200 eye movement related potential (EMRP) could be an important neural marker of the differentiation between high- and low-value retail items. Similarly, Tyson-Carr et al. (2018) found a pronounced (more negative) frontal N2 to low-valued items. We (Goto et al., 2017) found that viewing a group of highly preferred (HP) consumer goods led to more positive N200, LPP and PSW amplitudes compared to viewing less preferred (LP) goods. These results are consistent with results showing that preferences for pictorial figures as well as food items could modulate electrophysiological activity as early as 150 ms after stimulus onset, and up to 800 ms (Harris et al., 2011; Tzovara et al., 2015). Further, Pozharliev et al. (2015) did report effects of preference for luxury goods on the LPP, and Ma et al. (2018) reported larger LPP amplitudes for prices that could lead to buying intentions. In addition, Bosshard et al. (2016) found more positive ERP amplitudes from 800 to 2000 ms post-stimulus onset for liked compared to disliked brands. Overall these results suggest that three categories of group-related ERPs (N200, LPP, and PSW) might be sensitive to consumer preferences, although the differences between studies indicate that further replications are needed to strengthen these conclusions.

In summary, the current study had two goals. The primary goal of this study was to use standard ERP methods to test the claim that neural activity related to a single retail product is indicative of whether it is preferred or not. More specifically, we aimed to estimate if using classical ERP methods was viable to reliably estimate whether single-item ERP activity could predict behavioral preferences. Building on previous research (Pozharliev et al., 2015; Telpaz et al., 2015; Goto et al., 2017), we focused on three specific ERPs with distinct functional meanings: the N200, the LPP and the PSW. We devised an experimental procedure that isolated SI-ERPs related to 12 single consumer items in a simulated shopping context, and we compared SI-ERPs of each of these items to the average ERP activity of the six most highly preferred (HP) products and to the average of the six least preferred (LP) products. This procedure shares analogies with a situation in which neural activity related to a new product would be compared to neural activity related to a basket of “old” products for which preference levels are known. If SI-ERPs are accurate predictors of behavioral preferences, we expected that the amplitudes of SI-ERPs from items with high levels of behavioral preference should conform to two criteria. First, they should be larger than the averaged ERPs of a group of LP products Second, they should be equal or larger than the averaged ERPs of a group of other HP items. We predicted the converse pattern for SI-ERPs from items with low levels of behavioral preference. This approach allowed us to compute discrimination success scores for SI-ERPs across different ERP time windows, which enabled us to derive probabilities of committing errors when SI-ERPs are used to infer information about behavioral preferences for consumer goods. We were then able to test the main hypothesis of this study: If SI-ERPs are accurate predictors of behavioral preferences, then the probability of accurate predictions should be higher than the chance level.

The secondary goal of this study was to replicate Goto et al.’s (2017) results, and in particular the results showing that late group-related ERP positivities (LPP and PSW) can index consumer preferences. For this latter objective, we examined group-related ERP activity, averaging ERPs across different products separated in groups of HP vs. LP items.

## Materials and Methods

### Participants

Forty right-handed adults (27 females; mean age = 21.75, *SD* = 3.59) with no history of neurological or psychiatric disorders participated in this experiment. Our sample size is compatible with previous research (Pozharliev et al., 2015; Goto et al., 2017) and we did not use any stopping rule. From this initial sample, four participants were excluded because they did not have enough artifact-free trials (16) in at least one of the 12 products. This criterion was used to make sure that all ERP waveforms had an acceptable signal-to-noise ratio (SNR) (Luck, 2005). The final sample had 36 participants (25 females, mean age = 21.86, *SD* = 3.72). Participants were recruited from the student population of a foreign campus of an Australian University (Monash University) located in a large Asian urban center, the greater Kuala Lumpur metropolitan area (seven million inhabitants) in Malaysia. They were all fluent in English and they had all lived in Malaysia for more than a year. The Ethics committee of Monash University approved the study and all participants signed an informed consent before taking part in the experiment.

### Stimuli

During EEG recording, participants were shown a series of images depicting 12 consumer goods. Each of these goods were shown 30 times in three sets of 10 different pictures in order to maximize the SNR while mitigating potential habituation effects (Ravden and Polich, 1998). Therefore, we used color digital pictures of 12 products selected from a larger set of 180 pictures of retail items commonly found in outlets familiar to our sample of participants. These 12 products were selected from a price range of 21–40 Malaysian Ringgits in order to obtain a set of products that had a homogenous and affordable price level. In addition, care was taken to select a variety of products that were easily recognizable and available in local shops and retailers, and products from similar brands were not included. On the basis of the behavioral choice task used in the present study (see the *Behavioral Paradigm* section), we were able to assign a preference score to each product and separate them into a HP and a LP groups of products on the basis of a median split. Products were also ranked on the basis of their behavioral scores from #1 (the most preferred) to #12 (the least preferred product). HP and LP groups contained six products each. We chose this approach in order to make sure that behavioral preferences attached to these products were specific to the sample of the present study. These products were earphones, a box of ice cream, a box of Belgian chocolates, a 16G flash drive, a water bottle, a 5 kg bag of rice (HP products), a thermometer, a pack of 24 cans of a local drink, a set of four mugs, a shoe rack, a mosquito killer, and a calculator (LP products). As indicated in the Results section, products in the HP group were significantly more preferred than products in the LP group at *p* < 0.001 (more details are available in the Behavioral Results section). The average price of HP was 31.51 RM (*SD* = 4.87) and of LP was 29.73 RM (*SD* = 5.69) (approximately 7.51 and 7.09 USD, respectively). These products were actually bought by the researchers, and pictures of these items were taken 10 times from different angles. One picture was taken from a top (vertical) position, another from a side (horizontal) position, andthe others from oblique positions. For each oblique position, products were turned 45-degrees in a clockwise direction, resulting in eight pictures. All pictures were taken under similar lighting conditions with an Olympus camera. We provide example digital pictures of all the products and of a product taken from different angles in the supplementary section (Supplementary Figures 1, 2). In addition, we verified that HP and LP pictures did not vary according to low-level picture properties known to affect the ERPs used in this study (see Supplementary Section). Furthermore, given that a growing body of literature suggests that visual saliency plays a role in visual stimuli perception (Pedale and Santangelo, 2015; Santangelo et al., 2015; see for a review Santangelo, 2015), we also verified whether this variable influenced behavioral choice. We found that correlations between visual saliency and choice were all non-significant (see Supplementary Section for more details). This finding indicates that the rankings of our products on the basis of behavioral preferences were not influenced by potential systematic differences in visual saliency between HP and LP products.

### Behavioral Paradigm

Participants sat in a comfortable chair at approximately 80 cm from a 22” monitor on which the stimuli were displayed. E-Prime 2.0 (Psychology Software Tools, Pittsburgh, PA, United States) was used to display the stimuli on the screen. The experiment was separated into three phases. The 1st phase aimed at familiarizing participants with the stimuli. Participants were shown all the product pictures for the first time in order to prevent effects due to difficulties understanding or recognizing pictures of the products used in the subsequent phases of the experiment. In this phase, participants saw a fixation (black cross on white screen) displayed for 800 ms. Then, an image of one of the 12 products was shown for as long as participants wanted to see it. This procedure was repeated for all the 12 products. The 2nd phase was the viewing task. In this phase, 10 different pictures of each one of the 12 products were presented in a fully randomized order. Each picture was presented three times, which resulted in 360 trials. Participants were prompted to rest after each sequence of 90 trials.

**FIGURE 1:**
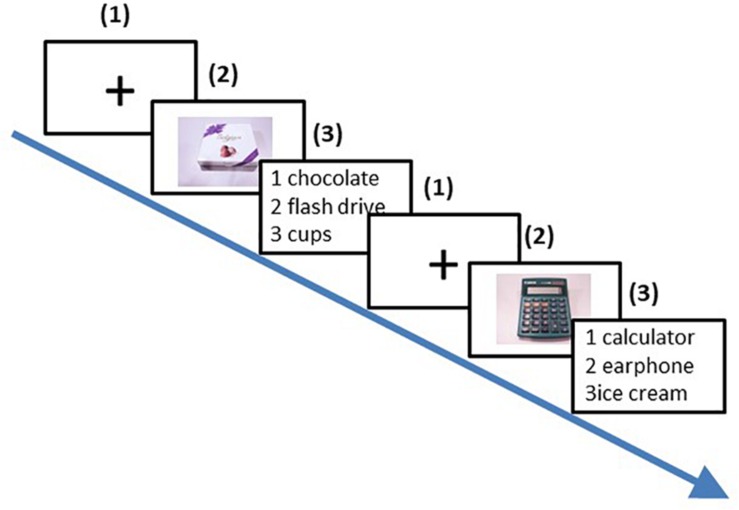
Viewing task procedure (Two trials). (1) A fixation screen was displayed for a random duration (800-1, 700 ms); (2) A picture of the product was presented for 3 s; (3) Participants indicated with a key press which description matched the product seen in slide (2). No time limit was imposed. This procedure was repeated for every picture of every product, resulting in 360 trials.

Each trial included three stages (depicted in Figure 1): First, participants saw a fixation displayed for a random duration (800–1700 ms). Second, an image of a product was shown on the computer screen for 3 s. Third, participants were asked to choose among three products descriptions the one that best matched the product they had just seen. The goal of this task was to ensure that participants paid attention to stimuli. There was no time limit for their responses on this task. The length of descriptions was matched with the target description ranging from one to four words. For example, if the product was a shoe rack, the three descriptions were as follows: (1) “shoes,” (2) “shoe rack,” and (3) “wardrobe.” Participants had to select the correct answer with a key press. No inaccurate responses were recorded, indicating that all products were easily recognizable for all participants. Throughout this phase, we recorded participants’ scalp EEG.

**FIGURE 2:**
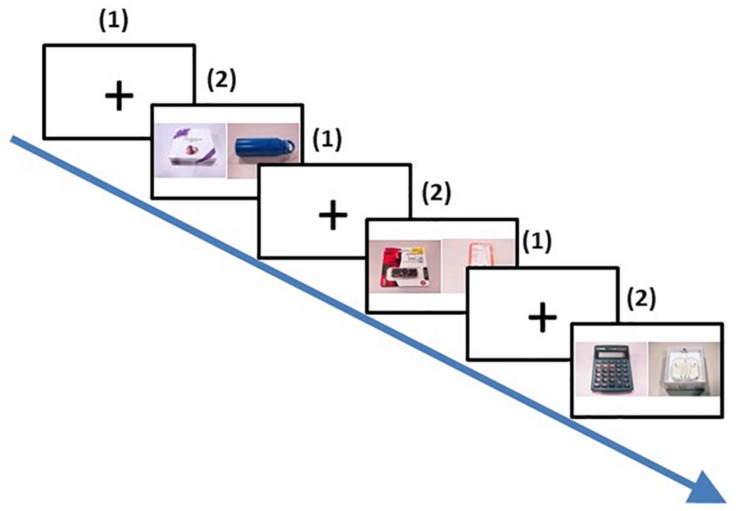
Trial procedure of the pairwise choice task (third phase). (1) A fixation screen was displayed for 1 second; (2) Two pictures of the two products were presented side by side for 3 s. Participants had to choose with a key press which of the the two products they preferred. This procedure was repeated for all possible pairs of the products and repeated 7 times, resulting in 462 trials.

The 3rd phase was the pairwise choice phase (Figure 2). Participants were shown on the screen pairs of the products side by side that they had seen in the previous phase. Each product was paired with other products without duplication, and each pair appeared only once within a block, resulting in 11(11+1)/2 = 66 pairs (trials) in a block. Each block was repeated seven times to minimize intra-item choice variability, which led to a total number of 462 trials. For every trial, the screen displaying the pair of products was preceded by a 1-s fixation screen, and lasted up to 3 s during which participants had to indicate with a key press which item they preferred out of the two displayed ones. The order of presentations and positions of pictures (left or right) were randomized for each repetition and across participants.

At the end of the experiment, participants saw the 12 pictures of each product again. Next, participants engaged in a “Willingness to pay” (WTP) task similar to a Becker-DeGroot-Marshak procedure (Becker et al., 1964), which could potentially lead to the actual purchase of the product. Specifically, for each product, they were asked to key in how much they were willing to pay from a virtual allocation of RM50, which was reset for every product. No time limit was set for this screen. If the price participants keyed in was more than a hidden price, which was randomly decided by a computer program for each product, then the product was considered as “successfully purchased.” After presenting all the products, a computer program randomly selected one of the successfully purchased products. This product and “cash savings,” (the RM50 allocation minus the hidden price for the product) were actually given to participants later. This approach was used to maximize the motivational engagement of participants in the task as this procedure linked the evaluation of the items to a real possibility of acquiring one of them (see Knutson et al., 2007; Schaefer et al., 2016). All of the aspects of the behavioral paradigm were explained to participants before the experiment, and their understanding was confirmed by a small quiz. The experiment lasted approximately 2 h.

### Electrophysiological Data Recording and Pre-processing

Each participant’s scalp EEG was recorded using 32 Ag/AgCl electrodes embedded in “Waveguard” purpose-made caps following the standard 10–20 system of electrode locations and an “ASALAB” amplifier (both manufactured by ANT Neuro, Enschede, Netherlands) at a sampling rate of 512 Hz (DC-138 Hz bandwidth). Impedance was kept below 10 kΩ, and a common average reference was used during recording. In order to minimize signal noise, EEG recordings took place in a custom-made Faraday cage (IAC Acoustics, Smithfield NSW, Australia). EEG pre-processing followed a standard procedure used in our previous work (e.g., Mushtaq et al., 2016; Goto et al., 2017), using the ERP module of BESA (Version 6.0, BESA GmbH, Gräfelfing, Germany). Data was converted offline to an average mastoids reference, filtered (0.01–30 Hz), segmented into epochs between 200 ms before and 3000 ms after the onset of the screen showing images of products (this event corresponds to the second step of the 2nd phase described above) and baseline corrected. Eye movements were corrected using a multiple source analysis method (Berg and Scherg, 1994; Ille et al., 2002) as implemented in BESA (“Surrogate method”). In addition, for each channel, epochs with a difference between the maximum and minimum voltage amplitude >120 μV and a maximum difference between two adjacent voltage points >75 μV were rejected (after eye movement artifact correction). After pre-processing, all 36 participants had at least 16 artifact-free trials for each of the 12 products (on average 25.46 artifact-free trials, range = 25.03–25.78) (see Participants section).

### ERP Quantification

The primary goals of our analysis of ERP data were twofold. First, we wanted to test whether SI-ERP activity could be used to make reliable inferences about the potential of specific goods to be preferred or not. Second we wanted to replicate our previous results showing that group-related ERPs could be modulated by consumer preferences (Goto et al., 2017). To ease the presentation of our data, the results referring to our second goal will be presented first. For these two goals, we needed to quantify three key ERP effects: the N200, the LPP and the PSW.

#### N200

The N200 was quantified using peak-to-peak scores. This is a recommended method to quantify temporally overlapping components, which is often the case in the P200/N200 complex (Luck, 2005; Hajcak et al., 2006; Osinsky et al., 2012; Mushtaq et al., 2013). On the basis of a visual inspection of our waveforms and previous literature (Kramer et al., 1995; Stefánsson and Jónsdóttir, 1996; Nieuwenhuis et al., 2004; Foti and Hajcak, 2008; Osinsky et al., 2012; Mushtaq et al., 2013, 2016; Telpaz et al., 2015; Decety et al., 2017), we extracted positive and negative peak amplitudes of each participant from two time windows: 130–230 ms (P200) and 200–400 ms (N200), respectively. Then, we subtracted P200 from N200 peaks, for every electrode in order to obtain the peak-to-peak measures of N200.

In order to make sure that our results were not specific to the peak-to-peak method, we also quantified the N200 using mean amplitudes extracted from a time window of 200–400 similar to the one used to quantify the peak-to-peak measure, and an *a priori* time window of 228–344 ms recommended in a recent meta-analysis of FRN/N200 effects (Sambrook and Goslin, 2015). Given that these methods led to similar results (i.e., a significant main effect of Preference, *p* < 0.001), we will report in this manuscript only the peak-to-peak results for the sake of conciseness. The results obtained with mean amplitudes can be consulted in the Supplementary Section and Supplementary Table 3.

#### Late Positivities

The LPP is usually quantified between 400 and 800 ms, whereas the PSW is usually observed after 800 ms, and can extend to 3 s or beyond (Diedrich et al., 1997; Foti and Hajcak, 2008; Hajcak and Olvet, 2008). Therefore, in line with our previous work (Goto et al., 2017), we separated late positivities into four consecutive 400-ms windows: 400–800, 800–1200, 1200–1600, 1600–2000, and we also quantified a longer time window from 2000 to 3000 ms (Foti and Hajcak, 2008; Hajcak and Olvet, 2008).

### Replication of Goto et al. (2017)

In order to replicate our previous results, we created a “Preference” factor, referring to the separation of the 12 products in two different categories according to their scores obtained in the behavioral pairwise choice task: The six most frequently chosen products were included in the HP category and the 6 least frequently chosen products were included in the LP category. Next, we created group-related ERP waveforms for the HP and LP conditions. For the N200, in order to perform statistical analyses, we extracted peak to peak scores from a sample of nine electrodes to cover Anterior, Central and Posterior sites, in left, midline and right hemispheres: F3, Fz, F4, C3, Cz, C4, P3, Pz, and P4. We then conducted a 2 (Preference) × 3 (Anterior-Posterior [AP]: Anterior vs. Central vs. Posterior) × 3 (Laterality [LAT]: Left, Midline vs. Right) repeated-measure analysis of variance (ANOVA). Sampling the scalp in two axes (anterior-posterior and left-right) is in line with standard practice in ERP research (e.g., MacKenzie and Donaldson, 2007; Bridger et al., 2009; Schaefer et al., 2011; Yick et al., 2015). For the late positivities, we extracted mean amplitudes and conducted ANOVAs identical to the ones employed for the N200 data, using the same array of electrodes across the five time windows described in the previous section. These ANOVAs were conducted separately for each time window. Since testing each effect repeatedly across the five different time windows of late positivities could inflate the false positive rate, we corrected the *p*-value of each test with the Bonferroni method. Greenhouse-Geisser corrections were applied when necessary, and results were considered significant at *p* < 0.05.

### Single-Item ERP Activity

In order to create SI-ERP waveforms, artifact-free trials of each product (on average 25.5 trials per participant) were combined and averaged into 12 ERP waveforms related to each of the 12 individual products. In order to ascertain if ERP activity relative to a given item could be predictive of whether it would be behaviorally preferred or not, we compared SI-ERPs of each single product to the group-related ERPs of HP and LP products, separately with pairwise contrasts for every time window. For every pairwise comparison, group-related ERPs did not include the SI-ERP of the product to which it was compared. For instance, when the SI-ERP of item #2 was compared to the group-related amplitude of the HP group of items (for any given time window), the HP group-related average was computed from items #1, #3, #4, #5, #6. In addition, given that we found that HP and LP averages were more strongly differentiated in anterior sites for the N200 and in posterior sites for late positivities, we focused on the anterior sites for the former and on the posterior sites for the latter. Specifically, we quantified SI-ERPs as the average of three fronto-central midline electrodes for the N200 (F3, Fz, and F4), and on the average of three midline parietal electrodes (P3, Pz, and P4) for the LPP and PSW. Averaging adjacent electrodes is often practiced and recommended to increase the stability of ERP data (Oken and Chiappa, 1986; Curran et al., 2006). Given that all comparisons across different time windows yielded 144 pairwise contrasts (12 items × 2 comparisons (HP and LP averages) × 6 time windows), we corrected *p*-values with a False Discovery Rate (FDR) procedure (Benjamini and Hochberg, 1995).

On the basis of the results obtained from a classical comparison between LP and HP averages (see section “Results”), and on the basis of our previous results (Goto et al., 2017), we predicted that for all time windows, behavioral preference should have a positive relationship with ERP amplitude. Furthermore, we tested whether each of the 144 pairwise comparisons conformed to criteria that could establish if SI-ERPs can reliably predict behavioral preference scores: If *x* refers to SI activity of a highly preferred item, and *y* refers to SI activity of a LP item; and if HP refers to the group-related ERP activity for items included in the HP group and LP refers to the group-related ERP activity for items included in the LP group; then, SI activity is informative of behavioral preference levels if:

(1) *x* ≥ HP AND *x* > LP;
  Or:
(2) *y* ≤ LP AND *y* < HP.

In the remainder of the article we will refer to these logical statements as the “discrimination criteria,” and they were tested with FDR-corrected pairwise tests described in Table 1. Single HP items were judged to be informative regarding behavioral preferences if they fulfill (1) and the same conclusion was applied to LP items that fulfill (2). Therefore, it is important to note that the < and > signs in the discrimination criteria refer to a statistical significance threshold of *p* < 0.05, FDR corrected.

**TABLE 1:** Single-item ERPs compared to the averaged ERPs of highly preferred (HP) and less preferred (LP) products.

**Rank**	**SI**	**LP**	**HP**	***p*-values against LP**	***p*-values against HP**	**SI**	**LP**	**HP**	***p*-values against LP**	***p*-values against HP**	**SI**	**LP**	**HP**	***p*-values against LP**	***p*-values against HP**
	
	**N200**					**400–800**					**800–1200**				
1	−**7.68**	−9.13	−7.93	0.002^*^	0.541	**5.37**	2.51	4.35	0.000^*^	0.020^*^	**2.29**	0.44	2.17	0.000^*^	0.727
2	−7.85	−9.13	−7.89	0.033	0.936	2.87	2.51	4.85	0.442	0.000^*^	0.87	0.44	2.45	0.341	0.000^*^
3	−8.92	−9.13	−7.68	0.545	0.010^*^	3.64	2.51	4.70	0.031	0.057	1.25	0.44	2.37	0.131	0.044
4	−**7.74**	−9.13	−7.91	0.003^*^	0.694	**5.42**	2.51	4.34	0.000^*^	0.019^*^	**2.87**	0.44	2.05	0.000^*^	0.070
5	−**7.03**	−9.13	−8.06	0.000^*^	0.023	**4.69**	2.51	4.49	0.000^*^	0.666	**2.65**	0.44	2.09	0.000^*^	0.220
6	−**8.09**	−9.13	−7.85	0.014^*^	0.584	**5.15**	2.51	4.40	0.000^*^	0.070	**3.19**	0.44	1.99	0.000^*^	0.016^*^
7	−8.18	−9.32	−7.89	0.031	0.455	**2.39**	2.54	4.52	0.749	0.000^*^	1.05	0.32	2.19	0.142	0.025
8	−8.94	−9.17	−7.89	0.649	0.036	3.44	2.33	4.52	0.052	0.057	**0.91**	0.35	2.19	0.302	0.021^*^
9	−8.19	−9.32	−7.89	0.012^*^	0.560	**1.97**	2.62	4.52	0.135	0.000^*^	**0.41**	0.45	2.19	0.920	0.000^*^
10	−8.54	−9.25	−7.89	0.221	0.177	**0.96**	2.82	4.52	0.001^*^	0.000^*^	−**1.56**	0.85	2.19	0.000^*^	0.000^*^
11	−**10.50**	−8.85	−7.89	0.009^*^	0.000^*^	**2.75**	2.46	4.52	0.564	0.000^*^	1.16	0.30	2.19	0.030	0.027
12	−**10.42**	−8.87	−7.89	0.000^*^	0.000^*^	3.57	2.30	4.52	0.007^*^	0.034	**0.72**	0.39	2.19	0.490	0.005^*^

	**1200–1600**					**1600–2000**					**2000–3000**				

1	**2.23**	−0.26	1.62	0.000^*^	0.200	**1.84**	−0.41	1.67	0.000^*^	0.722	**1.18**	−0.84	1.29	0.000^*^	0.842
2	0.37	−0.26	1.99	0.309	0.002^*^	0.77	−0.41	1.89	0.032	0.030	−0.02	−0.84	1.53	0.213	0.012^*^
3	**1.50**	−0.26	1.77	0.003^*^	0.594	**1.47**	−0.41	1.75	0.001^*^	0.609	**1.70**	−0.84	1.19	0.001^*^	0.445
4	**2.23**	−0.26	1.62	0.000^*^	0.271	**2.22**	−0.41	1.60	0.000^*^	0.316	**1.75**	−0.84	1.18	0.001^*^	0.404
5	**1.61**	−0.26	1.74	0.001^*^	0.783	**1.59**	−0.41	1.72	0.000^*^	0.785	**1.11**	−0.84	1.30	0.001^*^	0.742
6	**2.40**	−0.26	1.59	0.000^*^	0.163	**2.32**	−0.41	1.58	0.000^*^	0.137	**1.91**	−0.84	1.14	0.000^*^	0.211
7	0.67	−0.44	1.72	0.045	0.057	**0.43**	−0.58	1.70	0.073	0.019^*^	0.83	−1.17	1.27	0.001^*^	0.398
8	**0.37**	−0.38	1.72	0.210	0.012^*^	0.38	−0.57	1.70	0.151	0.040	−0.29	−0.94	1.27	0.317	0.036
9	−**0.54**	−0.20	1.72	0.526	0.001^*^	−**0.73**	−0.35	1.70	0.515	0.000^*^	−**1.40**	−0.72	1.27	0.276	0.000^*^
10	−**2.11**	0.11	1.72	0.000^*^	0.000^*^	−**2.13**	−0.07	1.70	0.000^*^	0.000^*^	−**2.68**	−0.47	1.27	0.001^*^	0.000^*^
11	**0.07**	−0.32	1.72	0.347	0.002^*^	−**0.51**	−0.39	1.70	0.832	0.000^*^	−**1.24**	−0.75	1.27	0.369	0.000^*^
12	**0.01**	−0.31	1.72	0.529	0.007^*^	**0.07**	−0.51	1.70	0.293	0.007^*^	−**0.23**	−0.96	1.27	0.182	0.016^*^

*P*-values are not corrected. ^*^ indicates significant differences after the correction by False Discovery Rate (FDR). SI-ERP values that conform to the discrimination criteria are printed in bold (See section “Materials and Methods”).

Intuitively, the discrimination criterion (1) reflects two simple principles: First, if SI-ERPs are accurate predictors of behavioral preference, then a highly preferred product (in behavioral terms) should have a significantly higher (*p* < 0.05) ERP amplitude than LP products. Second, if SI-ERPs are accurate predictors of behavioral preference, then an HP product (*x*) should have ERP amplitudes that are not significantly lower than other products that are also highly preferred. This criterion predicts that HP products should have equal amplitudes than other HP products but it also allows for potential cases in which HP products with exceptionally high levels of behavioral preference would have higher amplitudes than other HP products.

For instance, consider *x* as item #5 for the 400–800 window. Its SI-ERP amplitude is equivalent to 4.69. FDR-corrected comparisons in Table 1 indicate that this value is significantly higher than the LP group-related amplitude (2.51), and it is not significantly different from the HP group-related amplitude (4.49). In this case, discrimination criterion (1) is fulfilled and this specific SI-ERP can be said to be consistent with its corresponding behavioral preference score.

The overall predictive accuracy rate of SI-ERPs in the current study was inferred from the number of SI values that fulfilled the discrimination criteria, divided by the total number of SI values across all time windows and products. In addition, we also recalculated our results using a Bayesian inference framework combined with Monte-Carlo Markov Chain modeling. These additional analyses revealed similar results (i.e., a better accuracy for LPP and PSW compared to N200), and they are included in the Supplementary Section. The data of the results reported in this paper as well as in the Supplementary Section are available upon direct request to the corresponding author.

## Results

### Behavioral Preference Data

In the pairwise choice task, each product could appear 11 times. Since all the pairs were repeated seven times, the most preferred product could be chosen 77 times at most. Six individual HP items were chosen 60.75 (*SD* = 16.16), 59.64 (*SD* = 13.14), 52.56 (*SD* = 18.35), 51.81 (*SD* = 14.71), 43.67 (*SD* = 15.14), and 37.94 (*SD* = 18.92) times, respectively, and HP items were chosen on average 51.06 times (*SD* = 4.38). The 6 individual LP items were chosen 32.44 (*SD* = 15.31), 32.11 (*SD* = 17.70), 24.92 (*SD* = 15.13), 23.08 (*SD* = 17.75), 19.47 (*SD* = 18.29), and 16.44 (*SD* = 11.21) times, respectively. The average was 24.75 times (*SD* = 3.78). We also computed inter-subject correlations on the number of times each item was chosen. This analysis yielded 630 correlation coefficients with an average of *r*_mean_ = 0.450 (*r_*SD*_* = 0.271), which was significantly different from 0, *t*(629) = 41.07, *p* < 0.001. This finding indicates a significant level of behavioral inter-subject consistency between participants. Finally, we measured how consistently each participant chose one item over the other across the seven repetitions of each pair of products. The average number of pairs in which participants changed their choice more than twice was 6.22 (*SD* = 3.88) out of 66 pairs (9.43%). This finding indicates that participants showed clear, coherent preferences for more than 90% of the pairs. Finally, a paired-samples *t*-test revealed a significant difference, *t*(35) = 19.79, *p* < 0.001, between averaged HP items and LP items. As expected, we also observed a strong correlation between performance in the pairwise choice task and the WTP task (Spearman’s *r* = 0.73, *p* = 0.008).

### Brain Potentials

#### Replication Attempt of Goto et al. (2017)

##### N200

**FIGURE 3:**
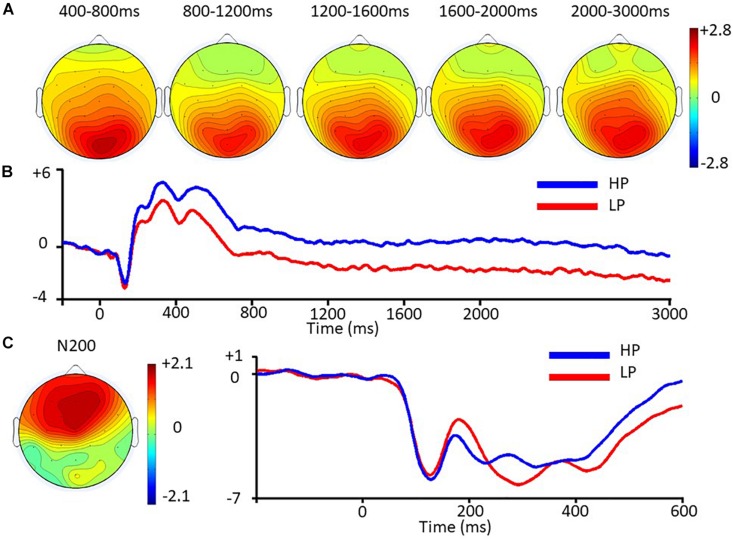
ERPs as a function of behavioral preferences. **(A)** Left: Two-dimensional scalp map plotting contrasts of late positivities between HP and LP. **(B)** ERP waveforms from Pz time-locked to the onset of pictures displaying consumer goods (second phase) separated by behavioral preferences. Amplitude in microvolts is on the y axis and time in milliseconds on the x axis. **(C)** Left: Two-dimensional scalp maps plotting contrasts of N200 peak to peak amplitudes between HP and LP. Right: ERP waveforms from Fz time-locked to the onset of pictures displaying consumer goods (second phase) separated by behavioral preferences. Amplitude in microvolts is on the y axis and time in milliseconds on the x axis.

Analyses on peak-to-peak scores yielded a significant main effect of Preference, *F*(1.00, 35.00) = 17.10, *p* < 0.001, η_*p*_^2^ = 0.328 and significant interactions with AP, *F*(1.74, 61.06) = 7.20, *p* < 0.001, η_*p*_^2^ = 0.171, and with LAT, *F*(1.85, 64.60) = 3.26, *p* = 0.049, η_*p*_^2^ = 0.085. Further analyses revealed that HP elicited more positive amplitudes than LP in all sites, *p*s < 0.022. The effect sizes of Preference were largest in Anterior sites (η_*p*_^2^ = 0.424), followed by Central (η_*p*_^2^ = 0.270) and Posterior sites (η_*p*_^2^ = 0.143). Furthermore, we observed that Preference effects were very large in Left and Midline sites, and followed by Right sites (η_*p*_^2^ = 0.342, 0.337, 273, respectively). See Figure 3C which shows a scalp map for N200 and waveforms from −200 to 600 ms on Fz. Analyses performed using two alternative methods of quantification (mean amplitudes on a 200–400 time window or on an *a priori* 228–344 time window) also yielded a significant main effect of Preference, indicating that our results on the N200 were not specific to the peak-to-peak method (see Supplementary Materials).

##### LPP

In the 400–800 ms time window, an interaction between Preference and AP was observed, *F*(1.41, 49.27) = 29.93, *p* < 0.001, η_*p*_^2^ = 0.461. Simple effects of Preference were significant in all sites (*p*s < 0.001), although effect sizes appeared to be larger in Posterior sites compared to Central and Anterior sites (η_*p*_^2^ = 0.637, 0.520, 279).

##### PSW

The main effect of Preference was significant for all four time windows (all *F*s > 14.15, *p*s < 0.004, Bonferroni-corrected, η_*p*_^2^ > 0.28). Interactions with AP were also observed (all *F*s > 25.07, *p*s < 0.001, Bonferroni-corrected, η_*p*_^2^ > 0.41). For all time windows, the simple effects of Preference were significant at the Central and Posterior sites, *p*s < 0.006, Bonferroni-corrected, with HP eliciting more positive amplitudes than LP. The effect sizes in the posterior sites were consistently stronger in all the four time windows (η_*p*_^2^ > 0.49) than those in the Central sites (η_*p*_^2^ < 0.37). The effects of Preference in the Anterior sites were not significant, *p*s > 0.14, uncorrected, η_*p*_^2^ < 0.06. See Figure 3A for scalp maps of the late positivities and Figure 3B for waveforms from −200 to 3000 ms on Pz. Similar effects were obtained using Bayesian inference methods (see the Supplementary Materials).

#### Single-Item ERP Activity

As explained in the Methods section, we created ERP waveforms related to each of the 12 products separately, and next we tested pairwise contrasts comparing ERPs of each single product to the group-related ERPs of HP and LP products, separately for every time window. Based on the results of the previous section, we focused on Anterior sites for the N200, and on Posterior sites for the late positivities. We report in Table 1 the uncorrected *p*-values of all the comparisons and indicate which ones survived a false discovery rate (FDR) correction for multiple comparisons (Benjamini and Hochberg, 1995). We also report in Table 2 whether each product fulfilled the discrimination criteria described in the Methods section, for each time window. A reanalysis of our data using a Bayesian inference approach also confirmed our results. These additional results are included in the Supplementary Section.

**TABLE 2:** Verification of statements related to discrimination *criteria*.

		**ERP time windows**					
		
**Discrimination criterion**	**Item rank**	**N200**	**400–800**	**800–1200**	**1200–1600**	**1600–2000**	**2000–3000**
1	1	TRUE	TRUE	TRUE	TRUE	TRUE	TRUE
1	2	FALSE	FALSE	FALSE	FALSE	FALSE	FALSE
1	3	FALSE	FALSE	FALSE	TRUE	TRUE	TRUE
1	4	TRUE	TRUE	TRUE	TRUE	TRUE	TRUE
1	5	TRUE	TRUE	TRUE	TRUE	TRUE	TRUE
1	6	TRUE	TRUE	TRUE	TRUE	TRUE	TRUE
2	7	FALSE	TRUE	FALSE	FALSE	TRUE	FALSE
2	8	FALSE	FALSE	TRUE	TRUE	FALSE	FALSE
2	9	FALSE	TRUE	TRUE	TRUE	TRUE	TRUE
2	10	FALSE	TRUE	TRUE	TRUE	TRUE	TRUE
2	11	TRUE	TRUE	FALSE	TRUE	TRUE	TRUE
2	12	TRUE	FALSE	TRUE	TRUE	TRUE	TRUE

*Discrimination criterion #1 (*x* ≥ HP AND *x* > LP) and criterion #2 (*y* ≤ LP AND *y* < HP). *x* refers to single-item activity of a highly preferred item, and *y* refers to single-item activity of a less preferred item. HP refers to the average ERP activity of the group of items included in the HP group, and LP refers to the average ERP activity of the group of items included in the LP group.*

There are 3 important observations to be noted from these data. First, Table 2 shows that 51 out of a total of 72 SI-ERP amplitude values conformed to our discrimination criteria, which leads to a 70.8% of predictive accuracy. Second, these figures vary according to which ERP effect is considered, as later positivities yielded a better performance: The implied probability of accurate predictions stands at 50% (6/12) for the N200 and 66.7% (8/12) for the LPP (400–800). For the PSW time windows, we found 66.7% (8/12) for 800–1200 ms, 83.3% (10/12) for both 1200–1600 and 1600–2000 ms, and 75% (9/12) for 2000–3000. The overall accuracy rate for the larger PSW time window (800–3000) stands at 77.1% (37/48) (see Supplementary Table 3). Third, SI activities of specific products may sometimes vary in ways that are contrary to expectations. For instance, product #2 is the second most preferred product on the behavioral choice task, but its SI-ERPs are consistently more negative than the group-related ERPs of the HP products in almost all time windows. Product #3 also seems to display the same counter-intuitive pattern for the N200, LPP and early PSW latencies. The counterintuitive pattern obtained for items #2 and #3 is visible from SI values in Table 1 and from Supplementary Figures 3, 4. Other items also seem to fail to conform to our expectations, depending on time windows and specific comparisons performed (e.g., products #12, #7, etc. See Table 2).

## Discussion

In this study, we had two main goals. First, our main goal was to verify if ERPs related to single consumer items could be used to reliably infer behavioral preferences. In other words, we wanted to examine whether SI-ERP activity for three specific brain potentials (N200, LPP, and PSW) could discriminate between items that have different levels of known behavioral preferences. Second, we wanted to replicate Goto et al.’s (2017) results showing that both early and late ERPs averaged across sets of different consumer goods (“group-related” activity) could be modulated by preference levels. Regarding our second objective, we successfully replicated Goto et al.’s (2017) results, as group-related activity for the N200, LPP and PSW were significantly modulated by levels of behavioral preference. Regarding our primary objective, when SI activity was compared to group-related activity for sets of HP and LP products, we found that 70.8% of our tests were consistent with criteria assessing the ability of SI activity to discriminate between items that have different levels of behavioral preferences. These results suggest that SI-ERPs’ overall predictive accuracy is above chance levels. However, our results have also shown that these scores vary according to specific ERP time windows. Late positivities (post-400 ERP effects) yielded relatively high predictive accuracy rates, whereas lower rates were attained by the frontal N200. Overall, although predictive accuracy rates of 70.8% can be considered as robust, they can also be seen as implying an overall 29.1% probability of reaching erroneous conclusions, which can increase to 50% if the N200 is used to compare products against average preferences. Therefore, our results suggest that caution is needed when behavioral preferences are inferred from SI-ERPs, as their predictive accuracy varies according to the type of ERPs considered, and according to the level of prediction error that researchers choose to tolerate.

The replication of our previous results (Goto et al., 2017) on group-related ERP activity for the N200, LPP, and PSW strengthen the point that both early and late ERP effects are related to behavior in a shopping context. As we elaborated previously (Goto et al., 2017), the relationship between consumer preferences and the set of ERPs evaluated in the current study (N200, LPP, and PSW) can be tentatively interpreted within the framework of a multi-stage process of selective attention toward consumer goods perceived as motivationally relevant. First, early ERPs could reflect a quick, pre-attentive orientation of attention toward preferred goods. Second, the LPP could reflect an overt attentional response linked to the conscious identification of the preferred goods. Finally, the PSW could reflect a facilitation of more elaborative cognitive processes. This explanation is consistent with existing models of motivated attention typically used to interpret similar ERPs associated with motivationally relevant stimuli (Schupp et al., 2006; Codispoti et al., 2007; Vuilleumier and Huang, 2009).

Although the results obtained with group-related ERPs have shown that the N200, LPP, and PSW were associated with consumer preferences, results obtained with SI-ERP activity indicate that they differed substantially in their predictive accuracy of behavioral preferences to single items. Specifically, the LPP and PSW yielded better predictive accuracy rates than the N200, which at times yielded predictive accuracy rates at the chance level. From the perspective of motivated attention models, these results may suggest that neural activity related to overt attentional processes can better predict behavioral preferences to single items than activity related to quick orienting processes, although we acknowledge that further research will be needed to confirm these conclusions. We discuss hereafter caveats and potential explanations regarding these findings.

First, in order to maximize SNR, we had to display every individual stimulus several times to our participants, while limiting the length of the experiment. These constraints led us to use only 12 individual products in our study, which indicates that our conclusions are limited by the scope of a relatively small universe of products. However, it is important to note that these products were not chosen arbitrarily (see section “Stimuli”) and they were affordable products taken from an array of different categories (food items, electronics, household items) that are representative of items commonly purchased by middle-income households in many countries. We do however acknowledge the need of more research to verify if the trends observed in the current study can be generalized to different sets of products.

Second, given that preference rankings were determined *a posteriori* by participants’ choices, content imbalances between HP and LP items can be expected. For instance, a higher number of food items can be found in HP compared to LP, and thus it can be argued that this imbalance may have biased the group-level HP-LP comparisons. However, Table 1 shows that food items in HP tend to be overall less positive than other HP items. Yet, we found robust main effects of Preference showing that HP amplitudes were more positive than LP amplitudes. If anything, the imbalance in food items would have worked against our main hypothesis, and the existence of main Preference effects despite this potential issue attests of the robustness of our results.

Third, given that some of the food items led to counterintuitive results (e.g., items #2 and #3), it could be argued that preference for food items are particularly difficult to predict with SI-ERPs, and that this potential phenomenon could account for most of the prediction errors that we observed in this study. This hypothesis is possible as preferences for food may be more context sensitive (e.g., whether or not participants are hungry). However, three important points need to be noted about this alternative explanation. Firstly, if these effects exist, they do not seem to be systematic as one of our food items (#6) had a 100% predictive accuracy in Tables 1, 2. Secondly, even if all food items were removed, the main conclusion would still be upheld, as the discrimination accuracy for later positivities occurring in an 800–3000 window would still be higher (87.5%) compared to the N200 (62.5%). Finally, there are only four food items in our set, and these numbers are not enough to make generalizations about the wider universe of food products available to consumers. Overall, given that our design relies on using a small number of items to maximize SI-related SNR, inferences about specific categories of objects can only be speculative at best. However, we do recognize the merit of considering specific categories of products in this type of research. It is an open question whether predictive accuracy varies depending on which specific category is investigated, and future research specifically designed to test this question is needed.

Fourth, given that the number of individual stimuli was constrained by our design, we were unable to investigate whether a series of product-level factors (e.g., familiarity, pleasantness, etc.) could modulate SI-ERPs and their predictive accuracy (c.f., Schoen et al., 2018). Future studies that systematically manipulate related stimuli-level or participant-level (e.g., SES) factors are needed to refine the knowledge about ERPs’ predictive power in CB.

Fifth, it could also be said that our results are limited by the scope of our choice of ERP components. Arguably, the N200, LPP, and PSW are not the only ERPs that could have been considered in the context of predicting consumer preferences. However, they are ERPs for which a relationship with consumer preferences has been shown by previous research (Pozharliev et al., 2015; Telpaz et al., 2015; Bosshard et al., 2016; Goto et al., 2017; Ma et al., 2018), and thus they are the most obvious choice for the current study. Another ERP component that could be interesting to test would be the Early Posterior Negativity (EPN, Junghöfer et al., 2006; Schupp et al., 2007), given its involvement with emotions and motivated attention processes (Schupp et al., 2006; Codispoti et al., 2007; Vuilleumier and Huang, 2009). However, given that the current study was not designed to test this specific ERP, we did not use an electrode array possessing the same extent and density of inferior temporal-occipital electrodes usually employed to evaluate this component (e.g., see Schupp et al., 2007). A natural evolution of this field of research would be to design studies that will assess how other ERPs can predict preferences, using SI-ERP activity.

Sixth, it could be argued that the utilization of a median split on behavioral scores to create HP and LP groups may have led to a problem of “fuzzy boundaries” between product groups (i.e., one could argue that the lowest and highest ranked items of HP and LP have relatively similar preference scores). However, we compared SI-ERPs to grand averages of HP and LP groups (rather than to other instances of SI-ERPs), which minimizes potential boundary issues caused by single items. Furthermore, we also recalculated our discrimination rates omitting boundary items (#6 and #7), and we obtained a total discrimination accuracy score of 71.7% which is approximately similar to our original result of 70.8%. Therefore, this finding suggests that our results were not affected by any potential problem of fuzzy boundaries.

Beyond any caveats, an important feature of our results is that a number of comparisons between SI-ERPs and group-related HP or LP ERPs were significant but in a direction opposite to expectations derived from their behavioral preference scores. This observation has important implications for the utilization of ERPs to predict behavioral preferences at the level of specific single consumer goods (rather than groups of diverse goods). As elaborated previously, in market research applications, it is often important to make predictive inferences at the level of single products, rather than from categories of products. If ERPs are to be used for such purpose, robust levels of inter-item consistency of the relationship between a given pattern of brain activity and behavioral preferences need to be established. However, neurophysiological measures can be modulated by content and context effects. For instance, previous research indicates that sometimes, equivalent processes of enhanced attention can be reflected by ERPs of different polarities (negative-going vs. positive-going) depending on stimulus content (Otten et al., 2007). Therefore, it is reasonable to assume that the high variation in attributes from one single product to the other (packaging, contents, price, etc.) can potentially be a source of significant variation in the nature of the neural activity related to the evaluation of these products, above and beyond preference levels. Averaging neural activity across different products while maintaining preference levels constant would tend to minimize inter-item variability.

Finally, our findings can lead to a number of recommendations regarding the interpretation of ERPs related to CB. First, if one decides to pursue the goal of predicting behavioral preferences of sets of various products regardless of differences between specific single items, then ERPs averaged across different consumer goods (“group-related” ERPs) seem to be reliable as indicated by our replication of Goto et al.’s (2017) results. This is consistent with the notion that computing ERPs averaged across different individual products could minimize potential variations in neural activity caused by inter-item content and context variability. Second, caution needs to be used when making inferences about behavioral preferences from SI-ERPs. SI neural activity on its own (i.e., in the absence of other measures of preference) should not be considered as a sufficiently reliable predictor of future behavioral preferences. However, SI-ERPs may be informative if they are used conjointly with other techniques (behavioral and psychophysiological) in order to improve measures of total predictive accuracy. Third, SI late ERP positivities in the latencies of the LPP and PSW seem to be more successful in discriminating items according to their levels of preferences, compared to the N200. However, predictive accuracy rates cannot be readily translated in applied settings, where the level of prediction errors tolerated by potential users can vary considerably.

In summary, we found that using SI-ERPs to predict preferences toward the items tested in the current study led to an overall predictive accuracy of 70.8%. However, we also found that prediction rates varied between different types of ERPs, as late positivities (LPP and PSW) were better predictors of preferences than the N200. In addition, our study also found that group-related ERPs could significantly differentiate between highly preferred and LP items for the N200, LPP and PSW. Further research will be needed to examine the potential reliability of ERPs to be applied in the field of CB research, such as the exploration of different sets of products, the consideration of specific categories of products and the manipulation of product features (e.g., Khushaba et al., 2012). Future research should also consider using alternative methods such as techniques involving machine learning methods (Pereira et al., 2009). The utilization of machine learning methods in ERP research is still rare (Rueda-Delgado et al., 2019), but it could provide valuable information in future studies tackling the relationship between neural activity and CB (e.g., Hakim et al., 2018).

## Data Availability

The datasets generated for this study are available on request to the corresponding author.

## Ethics Statement

This study was carried out in accordance with the recommendations of the Ethics committee of Monash University with written informed consent from all subjects. All subjects gave written informed consent before taking part in the experiment. The protocol was approved by the Ethics committee of Monash University.

## Author Contributions

NG and AS contributed conception and design of the study. NG, XL and DS organized the dataset. NG, XL, AH, KW, and LB performed the statistical analysis. NG and AS wrote the first draft of the manuscript. NG and XL wrote sections of the manuscript. All authors contributed to manuscript revision, read, and approved the submitted version.

## Conflict of Interest Statement

The authors declare that the research was conducted in the absence of any commercial or financial relationships that could be construed as a potential conflict of interest.
