# Supplementary material for: Can Brain Waves Really Tell If a Product Will Be Purchased? Inferring Consumer Preferences From Single-Item Brain Potentials
Source: Front Integr Neurosci. 2019 Jun 28;13:19. doi: 10.3389/fnint.2019.00019 (PMC6611214; doi:10.3389/fnint.2019.00019)
Supplement: Supplementary file 1 [file Image_1.pdf]

Supplementary Figure 1. Photographs of the 12 products used in the study, ordered by behavioural preference.

Supplementary Figure 2. Ten photographs of a product taken from different angles.

Supplementary Figure 3. Single-item ERP waveforms for the 12 products used in the study, at electrode Pz.

Supplementary Figure 4. Single-item ERP waveforms for the 12 products used in the study, at electrode Fz.

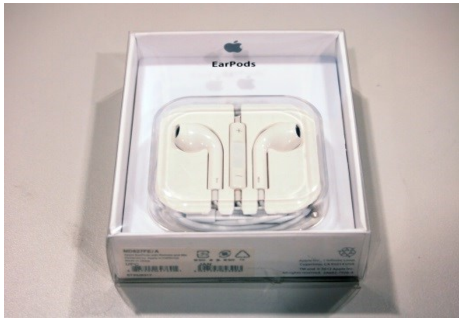

P1

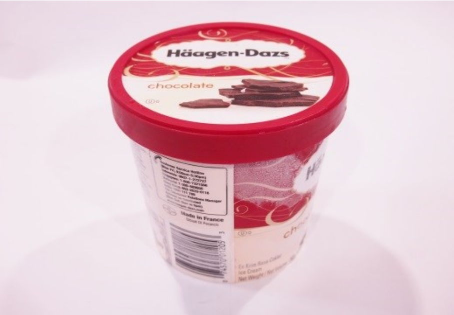

P2

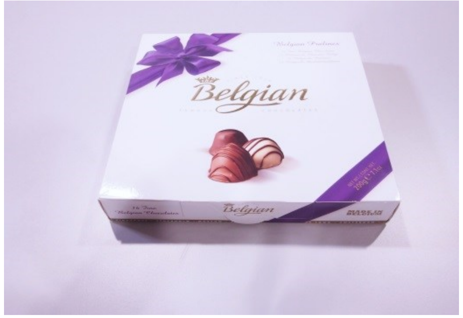

P3

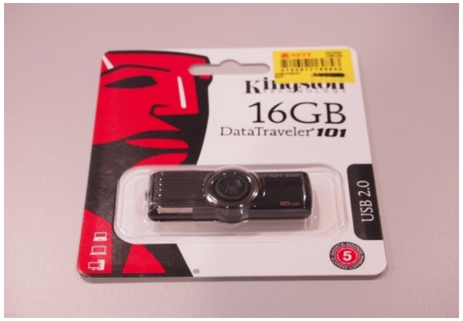

P4

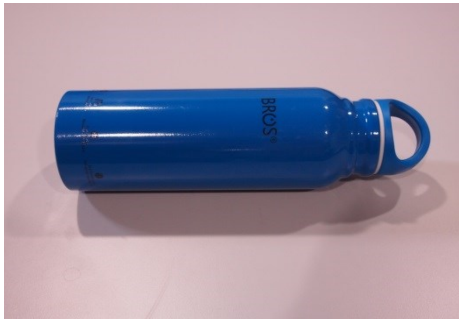

P5

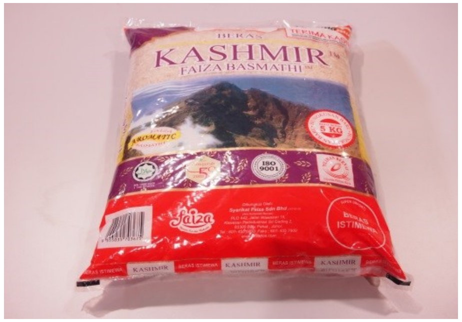

P6

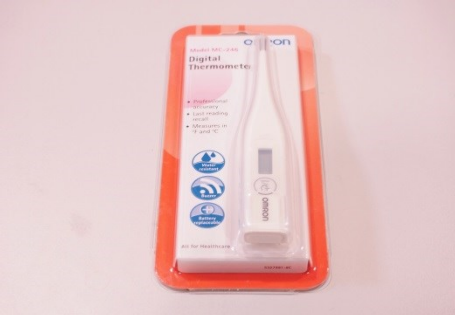

P7

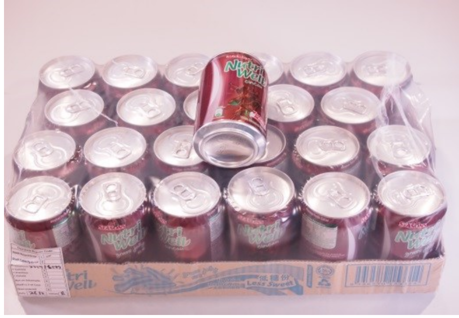

P8

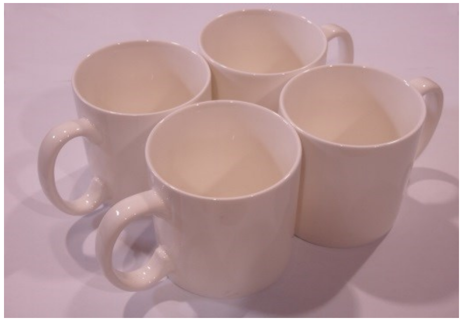

P9

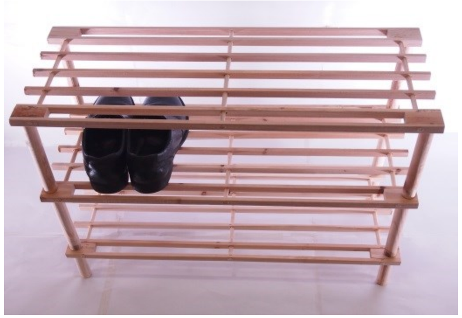

P10

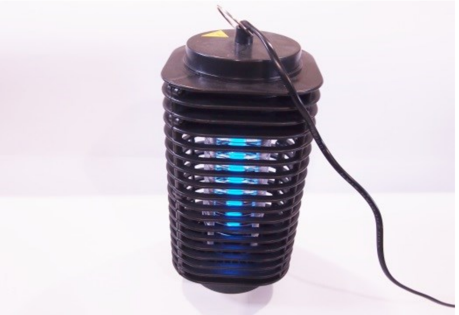

P11

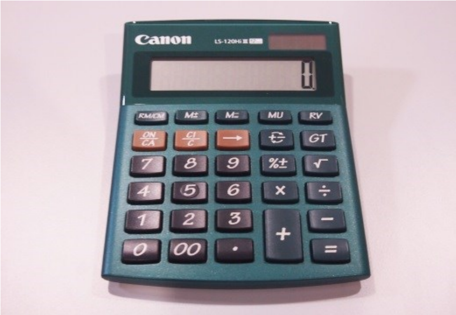

P12

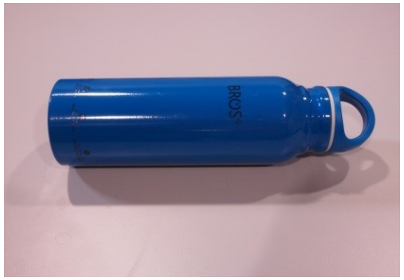
BROS®
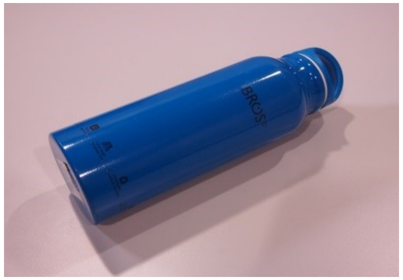
BROS®
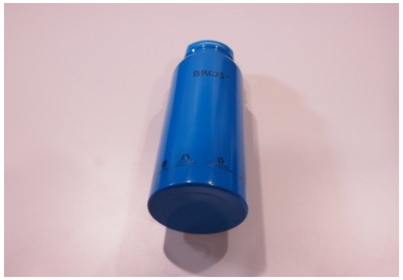
BROS®
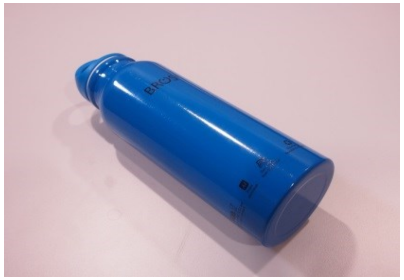
BROS®
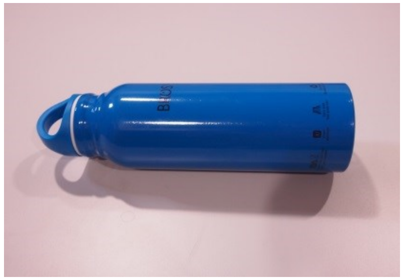
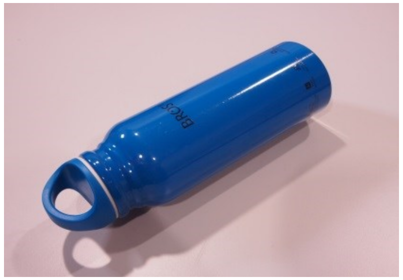
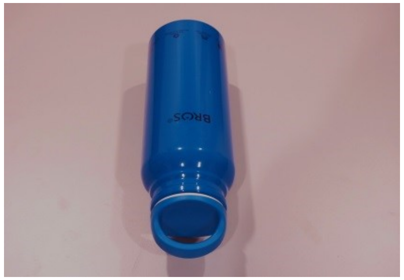
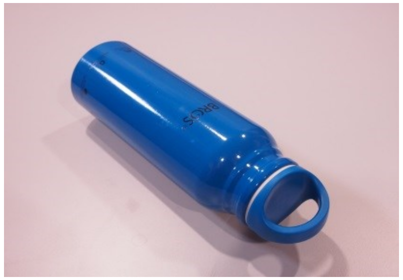
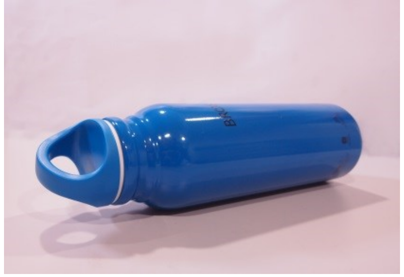
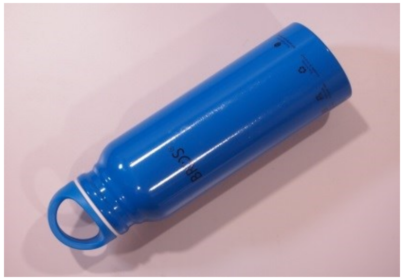

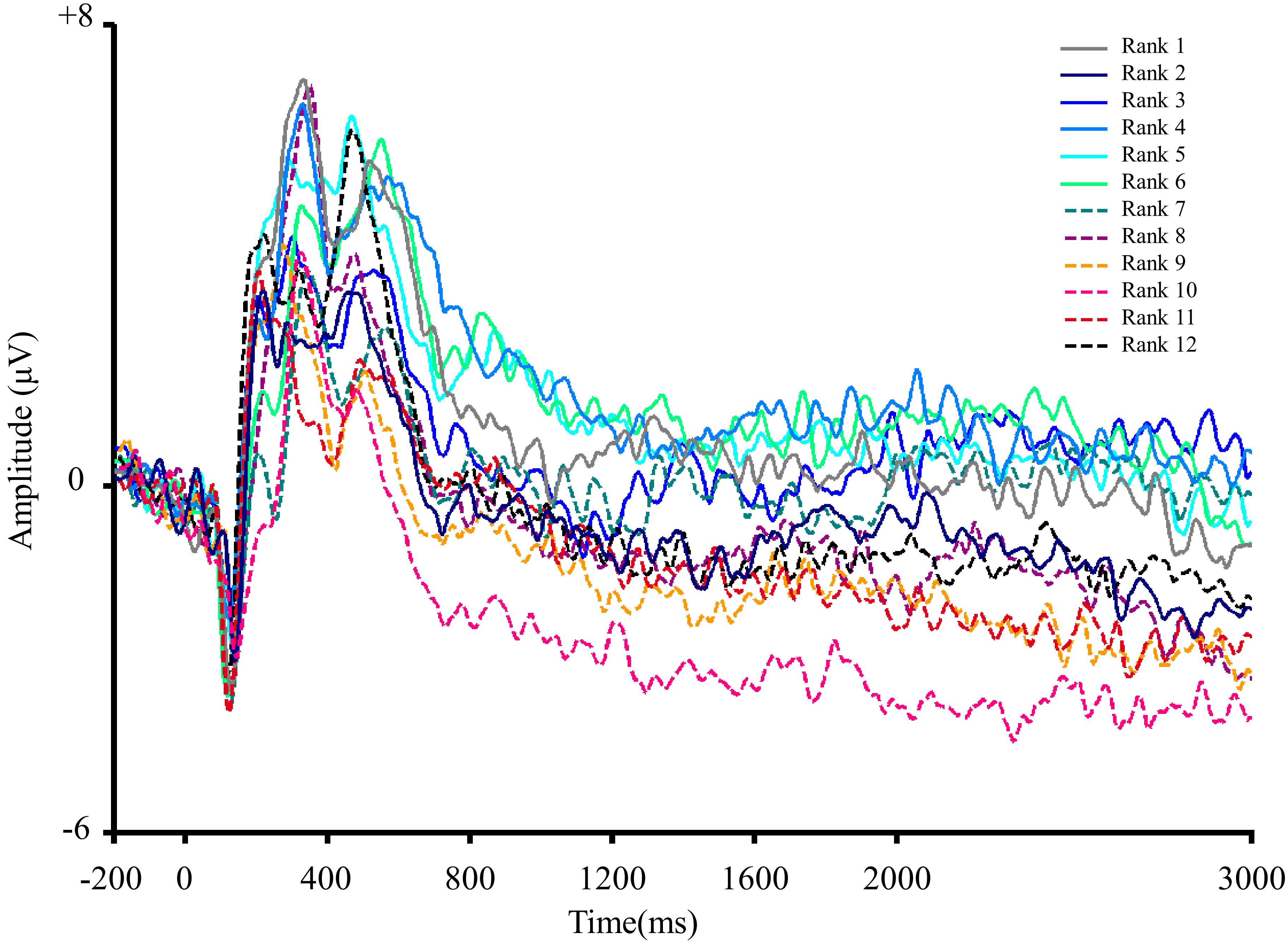

+8
0
-6
Amplitude (μV)
-200
0
400
800
1200
1600
2000
3000
Time(ms)
Rank 1
Rank 2
Rank 3
Rank 4
Rank 5
Rank 6
Rank 7
Rank 8
Rank 9
Rank 10
Rank 11
Rank 12

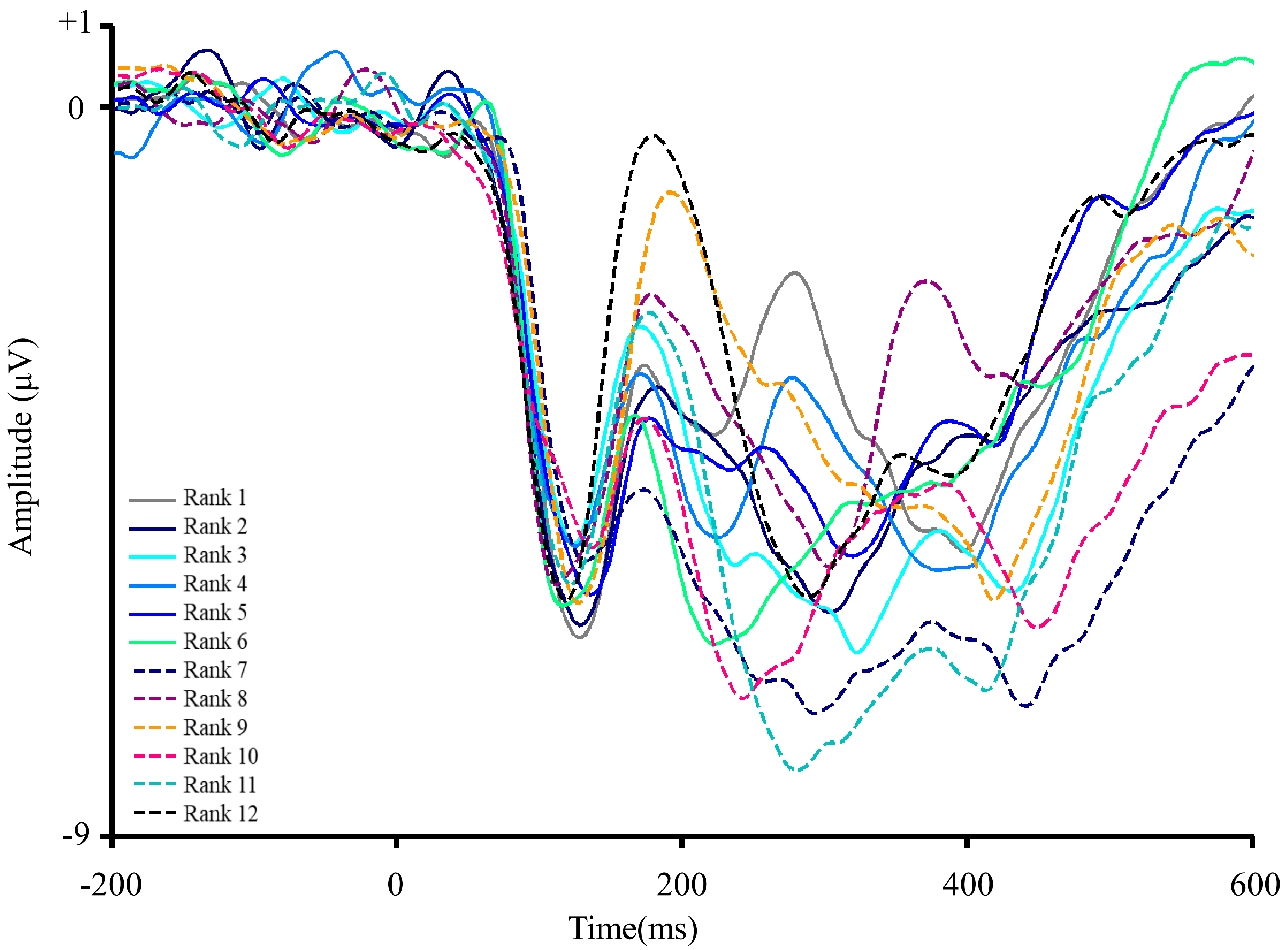

+1
0
-9
Amplitude (µV)
-200
0
200
400
600
Time(ms)
Rank 1
Rank 2
Rank 3
Rank 4
Rank 5
Rank 6
Rank 7
Rank 8
Rank 9
Rank 10
Rank 11
Rank 12
