# Supplementary material for: Can Brain Waves Really Tell If a Product Will Be Purchased? Inferring Consumer Preferences From Single-Item Brain Potentials
Source: Front Integr Neurosci. 2019 Jun 28;13:19. doi: 10.3389/fnint.2019.00019 (PMC6611214; doi:10.3389/fnint.2019.00019)
Supplement: Supplementary file 2 [file Table_1.pdf]

Low-level picture properties

We examined whether our pictures were not significantly different in low-level picture properties. Picture properties (brightness, contrast, spatial frequency, and visual complexity) were extracted for each of the 120 pictures using MATLAB (Bradley, Hamby, Löw, & Lang, 2007; Peli, 1990; Rosenholtz, Li, & Nakano, 2007). As described in our previous work (Watts, Buratto, Brotherhood, Barnacle, & Schaefer, 2014), brightness was defined as the mean red, green and blue intensity for each pixel averaged across all pixels in the picture. Contrast was obtained in two steps: First, the standard deviation of pixel intensities in each image column was computed; then the standard deviation across all image columns was computed. The latter was used as an index of contrast. Spatial frequency was obtained in three steps: First a power spectrum of the image was computed; then the frequency that split the area under the power spectrum in two equal halves was obtained for each row and for each column of the image; finally, these median-split frequencies were averaged across all rows and columns. This average was used as an index of the dominant spatial frequency in the picture. Visual complexity was estimated as the density of edge pixels (number of edge pixels divided by the total number of pixels in the image). Edge pixels were determined with Matlab's "canny" edge detector (low and high thresholds were set to 0.11 and 0.27, respectively). Edge density is associated with target detection time in visual search tasks and can be used as a measure of visual clutter or visual complexity (Rosenholtz et al., 2007). HP and LP pictures did not differ significantly in contrast, spatial frequency, and visual complexity (all *p*s > .10). However, we found that LP images were brighter than HP images ($p = .012$).

In order to examine whether the brightness of pictures is a good predictor of participants' preference choices, we did an analysis which is similar to what we did in Table 1.

More specifically, we compared the brightness of pictures of each item against its average of LP and HP items. Following the criteria that we used when examining the prediction accuracy of ERP amplitudes, we defined that brightness would predict preference if: 1a) the average brightness (10 pictures) of a single HP item is lower than the average brightness of LP items AND 1b) the average brightness of a single HP item is either higher or equal to the average brightness of the rest of HP items. For a single LP item, the expected direction of comparisons would be opposite; 2a) the average brightness (10 pictures) of a single LP item has to be higher than the average brightness of HP items AND 2b) the average brightness of a single LP item has to be either lower or equal to the average brightness of the rest of LP items. As it was the case for Table 1, the target item was not included for average when it was compared against its own preference category (Rank 1 versus HP items (Ranks 2, 3, 4, 5, and 6). With uncorrected p-values, we found that behavioural preferences of only 6 items out of 12 were predicted by pictures' brightness (see Supplementary Table 1 below). Thus, we verified that the ability of brightness scores to predict behavioural preference choice was at chance level. Furthermore, previous research shows that brightness does not have an effect on the ERPs targeted by this study, whereas visual complexity is a more important variable (Bradley et al., 2007; Schettino, Keil, Porcu, & Müller, 2016).

Supplementary Table 1

Single-Item pictures brightness compared to the averaged brightness of highly preferred (HP) and less preferred (LP) items.

	Brightness			Uncorrected p-values against LP	Uncorrected p-values agaist HP	Criteria fulfillment
Rank	SI	LP	HP			
1	174.97	190.01	182.97	.046	.600	**TRUE**
2	198.85	190.01	178.20	.404	.002	FALSE
3	201.15	190.01	177.74	.170	.000	FALSE
4	166.61	190.01	184.65	.000	.010	**TRUE**
5	164.62	190.01	185.05	.000	.003	**TRUE**
6	183.64	190.01	181.24	.910	1.000	FALSE
7	210.89	185.84	181.64	.000	.000	**TRUE**
8	180.28	191.96	181.64	.181	1.000	FALSE
9	169.50	194.12	181.64	.000	.109	FALSE
10	201.41	187.74	181.64	.082	.004	**TRUE**
11	201.68	187.68	181.64	.072	.004	**TRUE**
12	176.33	192.75	181.64	.023	1.000	FALSE

Saliency analyses of paired items

In order to examine whether salience of an item when presented in a pair was systematically related to which of a pair is chosen, we computed saliency of each pair using SaliencyToolbox 2.3 (Walther & Koch, 2006) and examined correlations between each item's preference score and the number of times each item had more salient part than its pair. We first put pictures of each item side by side and created a picture of every pair. Each item appeared both left and right, which resulted in 22 appearances of each item in different pairs (chocolate placed on the left side paired with the other 11 products and the same product placed on the right side paired with the other 11 products). This procedure resembles how each picture was presented to participants during the pairwise choice task. With these pictures, we used SaliencyToolbox 2.3 to determine in which area the most salient part would fall, either a product in the left, or in the right. The table below indicates the average number of times each product was chosen behaviorally (Choice: the biggest number it could take is 77), Ranking based on the behaviors (Rank: smaller numbers indicate higher ranks), and the number of times each product "won" the most salience when they are compared in a pair (Saliency: the biggest number it could take is 22).

Product	Choice	Rank	Saliency
pic¥cpri1.	60.75	1	2
pic¥cprk1	59.63889	2	15
pic¥cpra1	52.55556	3	21
pic¥cprg1	51.80556	4	11
pic¥cprb1	43.66667	5	18
pic¥cpre1	37.94444	6	9
pic¥cprd1	32.44444	7	9
pic¥cprc1	32.11111	8	7
pic¥cprf1.	24.91667	9	0
pic¥cprl1.	23.08333	10	14
pic¥cprj1.	19.47222	11	16
pic¥cprh1	16.44444	12	10

We took each pair as a subject and computed a correlation between the number of times an item on the left side was chosen behaviorally and whether an item on the left side had the most salient part, $r(132) = .103$, $p = .241$. We did the same for the number of times an item on the right was chosen and had the most salient part, $r(132) = .088$, $p = .318$. The mean of the two correlation coefficients was $r = .096$. These results seem that saliency did not have a systematic impact on participants' behavioral choices.

Note: Saliency is perfectly contingent on each other; if a left product has the most salient part, a right product of this pair does not. However, the number of times a left product was chosen does not automatically determine how many times a right of this pair was chosen. First, as we consider a pair of Products A and B and a pair of Products B and A (different positions) as different pairs, a same pair was repeated either 3 or 4 times. Second, participants may or may not choose a same product when its position is different. Third, some participants missed to respond a few (1% or less) trials. These randomness produces differences in the two correlations.

Supplementary analyses on mean amplitudes of 200-400 ms and 228-344 ms

Analyses performed on mean amplitudes (200-400) showed a significant main effect of Preference, $F(1.00, 35.00) = 16.19$, $p < .001$, $\eta p^2 = .316$ and significant interactions with AP, $F(1.48, 57.52) = 29.62$, $p < .001$, $\eta p^2 = .458$. Further analyses revealed that HP elicited more positive amplitudes than LP in Central and Posterior sites, $p = .017$, $\eta p^2 = .153$, $p < .001$, $\eta p^2 = .547$, respectively. The difference in Anterior site did not reach significance, $p = .108$, $\eta p^2 = .108$. Analyses performed on mean amplitudes obtained from an a priori of 228-344 time window (Sambrook & Goslin, 2015) revealed a significant main effect of Preference, $F(1.00, 35.00) = 25.632$, $p < .001$, $\eta p^2 = .423$ and significant interactions with AP, $F(1.45, 50.89) = 10.34$, $p = .001$, $\eta p^2 = .228$. Further analyses revealed that HP elicited more positive amplitudes than LP in all sites. The effect sizes of Preference were largest in Posterior sites ($\eta p^2 = .525$), followed by Anterior ($\eta p^2 = .305$) and Central sites ($\eta p^2 = .265$).

Bayesian analyses.

As mentioned in the main article, since product-to-average comparisons yielded 144 pairwise contrasts, we had to use a method to control for false positives. In addition, given that our primary goal was to evaluate the reliability of SI-ERPs in their potential to predict consumer behaviour, we needed robust estimates of differences between ERP amplitudes. Therefore, we tested paired *t*-tests between each product's SI-ERP amplitude and the HP and LP group-related average amplitudes using the Bayesian related sample inference framework available in IBM SPSS Statistics 25, involving a diffuse prior and a chain of 20,000 analysis samples obtained with Monte Carlo Markov Chain (MCMC) methods for each contrast. Bayesian inference methods can provide a robust control for false positives (Wetzels et al., 2011) and they can also provide estimates that are more resistant to distributional and outlier-related biases (Kanapathy, Khong, & Dekkers, 2014; Kruschke, 2013). We used a standard scheme that describes the

probability that the data could occur under the null hypothesis (Jeffreys, 1961): A Bayes factor (BF) of 1 provides evidence neither in favour of H1 nor H0, whereas BFs below 1 would provide anecdotal (0.3 to 1), substantial (0.1 to 0.3), strong (0.03 to 0.1), very strong (0.01 to 0.03) and decisive (BF<0.001) evidence in favour of H1. In order to obtain a protection against false positives, we decided to consider that ERP amplitudes are significantly different only if evidence in favour of H1 is at least of a substantial magnitude (<0.3) (Wetzels et al., 2011). In other words, BF<0.3 indicates that the data could have occurred 3.33 times (1/0.3) more likely under the alternative hypothesis (H1) compared to the null hypothesis (H0). Before we apply Bayesian methods to SI-ERPs , we report first a Bayesian analysis of the results of the replication of Goto et al.' (2017) .

Results of Group-related effects with Bayesian analysis.

In order to further test the reliability of this replication attempt, we performed HP-LP pairwise comparisons with Bayesian inference methods for every time window, on sites in which the Preference effect was the largest. As explained in the Methods section, an average of three frontal electrodes was used for the N200 and an average of parietal electrodes for all other time windows. This analysis revealed that all time windows yielded Bayes factors indicating "decisive" evidence in favour of a difference between HP and LP (all BFs ≤ 0.001). Overall, these results indicate that Goto et al.'s (2017) findings are replicated with robust methods.

Bayesian analysis of SI-ERPs.

We used exactly the same discrimination criteria explained in the Methods section, but we used a Bayesian framework to evaluate i they were fulfilled. We explain this approach in more details below:

If x refers to SI activity of a highly preferred item, and y refers to SI activity of a less preferred item; and if HP refers to the group-related ERP activity for items included in the HP

group and LP refers to the group-related ERP activity for items included in the LP group; Then, SI activity would be informative of behavioural preference levels if:

(1) $x \geq$ HP AND $x >$ LP;

Or:

(2) $y \leq$ LP AND $y <$ HP.

Single HP items were judged to be informative regarding behavioural preferences if they fulfilled (1) and single LP items were judged informative if they fulfilled (2). Fulfilment of (1) implies that x > LP is established by a BF inferior to 0.3. and that no x should be substantially (BF<0.3) inferior to HP. Fulfilment of (2) implies that $y <$ HP is established by a BF inferior to 0.3 and that y cannot be substantially higher than LP. It has to be noted that the fulfilment of our criteria when x is compared to HP or y to LP cannot be established by a simple confirmation of H0 (i.e. an absence of differences) because our criteria can be fulfilled if x (y) is *either* equal or larger (smaller) than HP (LP).

Supplementary Table 2 shows that 46 out of a total of 72 SI-ERP amplitude values conformed to our discrimination criteria, which leads to a 64% of predictive accuracy. Second, these figures vary according to which ERP effect is considered as later positivities seem to yield a better performance: The implied probability of accurate predictions stands at 41.7% (5/12) for the N200, 66.7% (8/12) for the LPP and 68.8% (33/48) for the PSW time windows (800-3000).

References

Bradley, M. M., Hamby, S., Löw, A., & Lang, P. J. (2007). Brain potentials in perception: Picture complexity and emotional arousal. *Psychophysiology*, *44*(3), 364–373. https://doi.org/10.1111/j.1469-8986.2007.00520.x

Goto, N., Mushtaq, F., Shee, D., Lim, X. L., Mortazavi, M., Watabe, M., & Schaefer, A. (2017). Neural signals of selective attention are modulated by subjective preferences and buying decisions in a virtual shopping task. *Biological Psychology*, *128*, 11–20. https://doi.org/10.1016/j.biopsycho.2017.06.004

Jeffreys, H. (1961). *Theory of probability* (3rd ed.). Oxford: Oxford University Press.

Kanapathy, K., Khong, K. W., & Dekkers, R. (2014). New product development in an emerging economy: Analysing the role of supplier involvement practices by using Bayesian Markov Chain Monte Carlo technique. *Journal of Applied Mathematics*, 1–12. https://doi.org/http://dx.doi.org/10.1155/2014/542606

Kruschke, J. K. (2013). Bayesian estimation supersedes the t test. *Journal of Experimental Psychology: General*, *142*(2), 573–603. https://doi.org/http://dx.doi.org/10.1037/a0029146

Peli, E. (1990). Contrast in complex images. *Journal of the Optical Society of America A*, *7*(10), 2032–2040. https://doi.org/10.1364/JOSAA.7.002032

Rosenholtz, R., Li, Y., & Nakano, L. (2007). Measuring visual clutter. *Journal of Vision*, *7*(2), 17. Retrieved from http://dx.doi.org/10.1167/7.2.17

Sambrook, T. D., & Goslin, J. (2015). A neural reward prediction error revealed by a meta-analysis of ERPs using great grand averages. *Psychological Bulletin*, *141*(1), 213–235. https://doi.org/10.1037/bul0000006

Schettino, A., Keil, A., Porcu, E., & Müller, M. M. (2016). Shedding light on emotional perception: Interaction of brightness and semantic content in extrastriate visual cortex. *NeuroImage*, *133*, 341–353. https://doi.org/https://doi.org/10.1016/j.neuroimage.2016.03.020

Walther, D., & Koch, C. (2006). Modeling attention to salient proto-objects. *Neural Networks*, *19*(9), 1395–1407. https://doi.org/https://doi.org/10.1016/j.neunet.2006.10.001

Watts, S., Buratto, L. G., Brotherhood, E. V, Barnacle, G. E., & Schaefer, A. (2014). The neural fate of neutral information in emotion-enhanced memory. *Psychophysiology*, *51*(7), 673–684. https://doi.org/10.1111/psyp.12211

Wetzels, R., Matzke, D., Lee, M. D., Rouder, J. N., Iverson, G. J., & Wagenmakers, E.-J. (2011). Statistical evidence in experimental psychology: An empirical comparison using 855 t Tests. *Perspectives on Psychological Science*, *6*(3), 291–298. https://doi.org/10.1177/1745691611406923
