# Supplementary material for: Can Brain Waves Really Tell If a Product Will Be Purchased? Inferring Consumer Preferences From Single-Item Brain Potentials
Source: Front Integr Neurosci. 2019 Jun 28;13:19. doi: 10.3389/fnint.2019.00019 (PMC6611214; doi:10.3389/fnint.2019.00019)
Supplement: Supplementary file 3 [file Table_2.pdf]

Supplementary Table 2

Single-Item ERPs compared to the averaged ERPs of highly preferred (HP) and less preferred (LP) products : Bayesian and frequentist analyses.

	N200							400-800							800-1200						
Rank	SI	LP	HP	p-values against LP	p-values against HP	Bayes Factor LP	Bayes Factor HP	SI	LP	HP	p-values against LP	p-values against HP	Bayes Factor LP	Bayes Factor HP	SI	LP	HP	p-values against LP	p-values against HP	Bayes Factor LP	Bayes Factor HP
1	**-7.68**	-9.13	-7.93	.002	.541	.066	6.410	**5.37**	2.51	4.35	.000	.020	.000	.538	**2.29**	0.44	2.17	.000	.727	.000	7.306
2	-7.85	-9.13	-7.89	.033	.936	.802	7.692	2.87	2.51	4.85	.442	.000	5.770	.002	0.87	0.44	2.45	.341	.000	4.953	.006
3	-8.92	-9.13	-7.68	.545	.010	6.462	.296	3.64	2.51	4.70	.031	.057	.771	1.281	1.25	0.44	2.37	.131	.044	2.505	1.039
4	**-7.74**	-9.13	-7.91	.003	.694	.100	7.146	**5.42**	2.51	4.34	.000	.019	.000	.508	**2.87**	0.44	2.05	.000	.070	.001	1.526
5	**-7.03**	-9.13	-8.06	.000	.023	.003	.602	**4.69**	2.51	4.49	.000	.666	.001	7.038	**2.65**	0.44	2.09	.000	.220	.002	3.686
6	-8.09	-9.13	-7.85	.014	.584	.388	6.648	**5.15**	2.51	4.40	.000	.070	.000	1.515	**3.19**	0.44	1.99	.000	.016	.001	.444
7	-8.18	-9.32	-7.89	.031	.455	.762	5.853	**2.39**	2.54	4.52	.749	.000	7.352	.002	1.05	0.32	2.19	.142	.025	2.649	.633
8	-8.94	-9.17	-7.89	.649	.036	6.993	.871	3.44	2.33	4.52	.052	.057	1.186	1.288	0.91	0.35	2.19	.302	.021	4.547	.551
9	-8.19	-9.32	-7.89	.012	.560	.348	6.519	**1.97**	2.62	4.52	.135	.000	2.551	.000	**0.41**	0.45	2.19	.920	.000	7.677	.014
10	-8.54	-9.25	-7.89	.221	.177	3.680	3.124	**0.96**	2.82	4.52	.001	.000	.023	.000	**-1.56**	0.85	2.19	.000	.000	.000	.000
11	**-10.50**	-8.85	-7.89	.009	.000	.259	.002	**2.75**	2.46	4.52	.564	.000	6.557	.011	1.16	0.30	2.19	.030	.027	.743	.691
12	**-10.42**	-8.87	-7.89	.000	.000	.010	.000	3.57	2.30	4.52	.007	.034	.210	.842	**0.72**	0.39	2.19	.490	.005	6.091	.155

	1200-1600							1600-2000							2000-3000						
Rank	SI	LP	HP	p-values against LP	p-values against HP	Bayes Factor LP	Bayes Factor HP	SI	LP	HP	p-values against LP	p-values against HP	Bayes Factor LP	Bayes Factor HP	SI	LP	HP	p-values against LP	p-values against HP	Bayes Factor LP	Bayes Factor HP
1	**2.23**	-0.26	1.62	.000	.200	.000	3.418	**1.84**	-0.41	1.67	.000	.722	.001	7.233	**1.18**	-0.84	1.29	.000	.842	.005	7.594
2	0.37	-0.26	1.99	.309	.002	4.613	.062	0.77	-0.41	1.89	.032	.030	.793	.752	-0.02	-0.84	1.53	.213	.012	3.590	.331
3	**1.50**	-0.26	1.77	.003	.594	.106	6.697	**1.47**	-0.41	1.75	.001	.609	.048	6.762	**1.70**	-0.84	1.19	.001	.445	.020	5.794
4	**2.23**	-0.26	1.62	.000	.271	.002	4.225	**2.22**	-0.41	1.60	.000	.316	.009	4.677	**1.75**	-0.84	1.18	.001	.404	.033	5.480
5	**1.61**	-0.26	1.74	.001	.783	.044	7.425	**1.59**	-0.41	1.72	.000	.785	.009	7.423	**1.11**	-0.84	1.30	.001	.742	.052	7.337
6	**2.40**	-0.26	1.59	.000	.163	.004	2.939	**2.32**	-0.41	1.58	.000	.137	.000	2.569	**1.91**	-0.84	1.14	.000	.211	.007	3.562
7	0.67	-0.44	1.72	.045	.057	1.052	1.288	0.43	-0.58	1.70	.073	.019	1.563	.503	0.83	-1.17	1.27	.001	.398	.035	5.432
8	0.37	-0.38	1.72	.210	.012	3.542	.345	0.38	-0.57	1.70	.151	.040	2.767	.956	-0.29	-0.94	1.27	.317	.036	4.715	.869
9	**-0.54**	-0.20	1.72	.526	.001	6.318	.027	**-0.73**	-0.35	1.70	.515	.000	6.239	.011	**-1.40**	-0.72	1.27	.276	.000	4.299	.007
10	**-2.11**	0.11	1.72	.000	.000	.001	.000	**-2.13**	-0.07	1.70	.000	.000	.012	.000	**-2.68**	-0.47	1.27	.001	.000	.023	.000
11	**0.07**	-0.32	1.72	.347	.002	4.968	.063	**-0.51**	-0.39	1.70	.832	.000	7.532	.002	**-1.24**	-0.75	1.27	.369	.000	5.190	.001
12	**0.01**	-0.31	1.72	.529	.007	6.333	.206	**0.07**	-0.51	1.70	.293	.007	4.451	.218	-0.23	-0.96	1.27	.182	.016	3.197	.444

Note: *P* -values are not corrected. Bold face indicates SI amplitudes that successfully predict behavioural preference scores, according to criteria #1 and #2 described in the Methods section ("Single-item ERP activity" subsection).
