# Supplementary material for: Can Brain Waves Really Tell If a Product Will Be Purchased? Inferring Consumer Preferences From Single-Item Brain Potentials
Source: Front Integr Neurosci. 2019 Jun 28;13:19. doi: 10.3389/fnint.2019.00019 (PMC6611214; doi:10.3389/fnint.2019.00019)
Supplement: Supplementary file 4 [file Table_3.pdf]

Supplementary Table 3

Single-Item ERPs compared to the averaged ERPs of highly preferred (HP) and less preferred (LP) products : N200 mean amplitudes and 800-3000 extended time window.

Peak-to-peak N200

Rank	SI	LP	HP	p-values against LP	p-values against HP	Bayes Factor LP	Bayes Factor HP
1	**-7.68***	-9.13	-7.93	.002*	.541	0.066	6.410
2	-7.85	-9.13	-7.89	.033	.936	0.802	7.692
3	-8.92	-9.13	-7.68	.545	.010*	6.462	0.296
4	**-7.74***	-9.13	-7.91	.003*	.694	0.100	7.146
5	**-7.03***	-9.13	-8.06	.000*	.023	0.003	0.602
6	-8.09*	-9.13	-7.85	.014*	.584	0.388	6.648
7	-8.18	-9.32	-7.89	.031	.455	0.762	5.853
8	-8.94	-9.17	-7.89	.649	.036	6.993	0.871
9	-8.19	-9.32	-7.89	.012*	.560	0.348	6.519
10	-8.54	-9.25	-7.89	.221	.177	3.680	3.124
11	**-10.50***	-8.85	-7.89	.009*	.000*	0.259	0.002
12	**-10.42***	-8.87	-7.89	.000*	.000*	0.010	0.000

200-400 ms mean amplitudes

Rank	SI	LP	HP	p-values against LP	p-values against HP	Bayes Factor LP	Bayes Factor HP
1	**-3.64***	-5.03	-4.97	.001*	.007*	0.039	0.206
2	-4.75	-5.03	-4.75	.397	.996	5.418	7.732
3	-5.61	-5.03	-4.58	.147	.032	2.730	0.790
4	-4.52	-5.03	-4.80	.181	.533	3.182	6.377
5	-4.55	-5.03	-4.79	.300	.590	4.537	6.696
6	-5.43	-5.03	-4.61	.314	.046	4.673	1.072
7	**-6.36***	-4.76	-4.75	.002*	.001*	0.064	0.034
8	-3.66	-5.30	-4.75	.003*	.016*	0.085	0.440
9	-3.56	-5.32	-4.75	.001*	.008*	0.028	0.238
10	-5.59	-4.92	-4.75	.299	.136	4.531	2.567
11	**-6.54***	-4.73	-4.75	.000*	.000*	0.010	0.009
12	-4.46	-5.14	-4.75	.202	.541	3.446	6.423

228-344 ms mean amplitudes

Rank	SI	LP	HP	p-values against LP	p-values against HP	Bayes Factor LP	Bayes Factor HP
1	**-3.06***	-5.45	-5.11	.000*	.000*	0.000	0.017
2	-5.22	-5.45	-4.68	.513	.114	6.254	2.235
3	-5.89	-5.45	-4.55	.337	.011*	4.894	0.310
4	**-4.09***	-5.45	-4.90	.002*	.085	0.057	1.785
5	-4.76	-5.45	-4.80	.170	.938	3.035	7.709
6	-5.56	-5.45	-4.61	.788	.037	7.460	0.887
7	**-6.59***	-5.22	-4.77	.010*	.000*	0.280	0.018
8	-4.43	-5.65	-4.77	.029	.490	0.738	6.106
9	-3.56	-5.83	-4.77	.000*	.009*	0.004	0.253
10	-5.97	-5.35	-4.77	.372	.039	5.209	0.939
11	**-7.13***	-5.12	-4.77	.000*	.000*	0.005	0.001
12	-5.03	-5.54	-4.77	.393	.623	5.387	6.860

800-3000

Rank	SI	LP	HP	p-values against LP	p-values against HP	Bayes Factor LP	Bayes Factor HP
1	**1.34***	-0.76	1.24	.000*	.851	0.002	7.598
2	0.06	-0.76	1.50	.178	.011*	3.152	0.302
3	**1.15***	-0.76	1.28	.001*	.811	0.036	7.515
4	**1.84***	-0.76	1.14	.000*	.190	0.001	3.306
5	**1.19***	-0.76	1.27	.001*	.889	0.035	7.658
6	**1.98***	-0.76	1.12	.000*	.132	0.004	2.517
7	0.15	-0.94	1.26	.052	.054	1.194	1.237
8	-0.16*	-0.88	1.26	.176	.012*	3.116	0.338
9	**-1.32***	-0.64	1.26	.272	.000*	4.250	0.008
10	**-2.50***	-0.41	1.26	.000*	.000*	0.008	0.000
11	**-0.54***	-0.80	1.26	.565	.001*	6.563	0.027
12	-0.18*	-0.87	1.26	.199	.011*	3.411	0.303

Note: *P* -values are not corrected. Bold face indicates SI amplitudes that successfully predict behavioural preference scores, according to criteria #1 and #2 described in the Methods section ("Single-item ERP activity" subsection).
